# Emotional context can reduce the negative impact of face masks on inferring emotions

**DOI:** 10.3389/fpsyg.2022.928524

**Published:** 2022-09-23

**Authors:** Sarah D. McCrackin, Jelena Ristic

**Affiliations:** Department of Psychology, McGill University, Montreal, QC, Canada

**Keywords:** lower face occlusion, face masks, emotion recognition, emotional inference, emotional context, face feature occlusion

## Abstract

While face masks prevent the spread of disease, they occlude lower face parts and thus impair facial emotion recognition. Since emotions are often also contextually situated, it remains unknown whether providing a descriptive emotional context alongside the facial emotion may reduce some of the negative impact of facial occlusion on emotional communication. To address this question, here we examined how emotional inferences were affected by facial occlusion and the availability of emotional context. Participants were presented with happy or sad emotional faces who were either fully visible or partially obstructed by an opaque surgical mask. The faces were shown either within an emotionally congruent (e.g., “Her cat was found/lost yesterday afternoon”) or neutral (“Get ready to see the next person”) context. Participants were asked to infer the emotional states of the protagonists by rating their emotional intensity and valence. Facial occlusion by masks impacted the ratings, such that protagonists were judged to feel less intense and more neutral emotions when they wore masks relative to when their face was fully visible. Importantly, this negative impact of visual occlusion by mask was reduced but not fully eliminated when the faces were presented within a congruent emotional context. Thus, visual occlusion of facial emotions impairs understanding of emotions, with this negative effect of face masks partially mitigated by the availability of a larger emotional context.

## Introduction

With the onset of the COVID-19 pandemic, the world saw an unprecedented but necessary widespread adoption of face masks. While masks provide much needed protection again virus spread (Eikenberry et al., 2020; Leung et al., 2020; Prather et al., 2020), they also present challenges to visual social communication as they obstruct approximately 60–70% of the face parts needed for socioemotional messaging (e.g., Carbon, 2020; Mheidly et al., 2020; Molnar-Szakacs et al., 2021 for reviews). Faces are some of most important social stimuli we encounter, and humans readily utilize visible cues from faces to recognize emotions in others (Hugenberg and Wilson, 2013). It has long been demonstrated that facial expressions provide a quick and easy way to extract information about others’ emotional states (e.g., Ekman, 1999), with the ability to read these expressions associated with increased levels of several facets of overall social functioning (e.g., Leppänen and Hietanen, 2001; Addington et al., 2006) including prosocial behavior (Marsh et al., 2007), social approach (Williams et al., 2014), and empathy (Besel and Yuille, 2010).

Many social judgments are made from facial cues (e.g., Klapper et al., 2016). It is thus unsurprising that face masks have been shown to impact many of these judgments, including reducing perceived closeness (Grundmann et al., 2021), increasing perceived attractiveness (Hies and Lewis, 2022; Parada-Fernández et al., 2022) and either increasing (Cartaud et al., 2020; but see Grundmann et al., 2021) or decreasing (Biermann et al., 2021; Gabrieli and Esposito, 2021) perceived trustworthiness. The alterations in such second-order trait perception likely stem from the obstruction of the visual information from the lower face cues needed for basic processes that inform these judgments, such as emotion recognition (Carbon, 2020; Grundmann et al., 2021; Carbon and Serrano, 2021; Williams et al., 2021; Grenville and Dwyer, 2022; Kim et al., 2022; McCrackin et al., 2022a; Parada-Fernández et al., 2022). Indeed, emotion recognition performance for faces obstructed by face masks can decline from 10 to 45% depending on the emotional expression (Carbon, 2020; McCrackin et al., 2022a). That is, recognition of emotional expressions thought to have particularly diagnostic lower face features like disgust and anger (e.g., Ekman, 1999; Smith et al., 2005; Blais et al., 2012; Kret and de Gelder, 2012; Wegrzyn et al., 2017) is the most impacted by lower face occlusion while recognition of expressions with diagnostic upper face regions like fear and surprise is least impacted by lower face occlusion (Carbon, 2020; Carbon and Serrano, 2021; Williams et al., 2021; Grenville and Dwyer, 2022; Kim et al., 2022; McCrackin et al., 2022a).

While the impact of masks on basic emotion recognition is clear (Carbon, 2020; Grundmann et al., 2021; Carbon and Serrano, 2021; Williams et al., 2021; Grenville and Dwyer, 2022; Kim et al., 2022; McCrackin et al., 2022a), recognition of emotional expressions is typically situated within a broader emotional context (Wieser and Brosch, 2012). For example, one might perceive a smile alongside a joke being told or hearing good news being shared. In other words, making emotional inferences requires not only emotional information from faces (Baron-Cohen and Cross, 1992; Clark et al., 2008; Mier et al., 2010; Decety et al., 2015; Stewart et al., 2019) but also contextual information like emotional prosody, body language, prior knowledge, emotional understanding, and/or emotional context (Wieser and Brosch, 2012 for a review).

Here we sought to examine the role of emotional context in making emotional inferences from faces occluded by face masks. Can availability of emotional context ameliorate the negative impact of facial occlusion on emotional communication? To address this question, we asked participants to judge the emotions of protagonists who displayed happy and sad facial expressions and either wore a surgical mask or had their face visually unobstructed. Critically, on half of the trials, the protagonists were presented within a congruent emotional context—a written sentence describing a happy or sad event happening to the protagonist. In the other half of trials, the protagonists were presented within a neutral context—a written sentence informing participants to get ready for the next trial. On each trial, participants rated the intensity and valence of the protagonist’s emotion. Intensity refers to degree of arousal, while valence refers to the degree of pleasantness. The Circumplex theory of emotion (Russell, 1980) suggests that intensity and valence constitute two unique dimensions of affective experience. There is both behavioral and neural evidence to suggest that these dimensions can be dissociated (e.g., Anderson and Sobel, 2003; Kensinger and Corkin, 2004; Colibazzi et al., 2010). For example, strong positive valence can either be paired with high intensity in the experience of happiness or low intensity in the experience of serenity. Thus we decided to examine how facial obstruction by face masks impacted both aspects of affective perception. We also reasoned that using ratings of valence and intensity as in our previous work (see also McCrackin and Itier, 2021; McCrackin et al., 2022a) would avoid potential ceiling effects that may occur from the utilization of forced choice paradigms typically used in emotion recognition given that here we did not only present facial emotional expressions, but also emotional sentences with clear emotional content.

Following from past work (e.g., McCrackin et al., 2022b), we expected to observe diminished (i.e., more neutral) ratings of the protagonists’ emotions when they wore masks. However, if this negative effect of facial obstruction was modulated by the availability of emotional context, we expected to find higher emotional ratings for faces wearing masks in conditions in which congruent context was provided relative to conditions in which no context was provided. Our data supported these predictions.

## Methods

This study was pre-registered.^1^ Anonymized and summarized data are available at https://osf.io/9bmr3/?view_only=e0871f7add364e378eccd9920f60d98b.

### Participants

Seventy undergraduate students participated for course credit and were included in the analysis (66 female, 3 male, 1 other; Mean age*:* 20.41, *SE* = 0.13).^2^ Sample size was pre-registered and determined with a conservative power analysis based on our previous work with face masks and emotion recognition (McCrackin et al., 2022a) and affective theory of mind (McCrackin et al., 2022b). Participants provided informed consent and the McGill University research ethics board approved the study.

### Apparatus and stimuli

**FIGURE 1:**
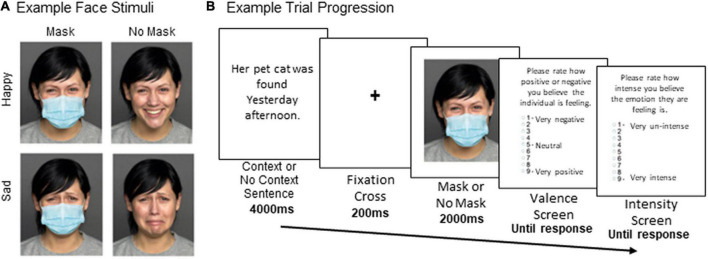
**(A)** Example face stimuli. **(B)** Example trial progression. In the Context condition, an emotional sentence congruent with the face emotion (either happy or sad) was provided at the start. In the No context condition, the sentence “Get ready for the next person to appear” was presented instead. Following the emotional face image, participants rated the intensity and valence of the protagonist’s emotional state using two separate scales (valence and intensity).

The experiment took place online *via* Testable^3^ with stimuli scaled to fit each participant’s personal computer screen. Sample face stimuli are shown in Figure 1A. Images of happy and sad face stimuli were obtained for 20 male and 20 female identities from the FACES (Ebner et al., 2010) and Karolinska Directed Emotional Faces (KDEF; Lundqvist et al., 1998) databases, which have independently validated that these images depict facial expressions with high recognizability (Goeleven et al., 2008; Ebner et al., 2010).^4^ For the Mask condition, a photograph of a surgical mask was applied to each face using Adobe Photoshop CS6 and scaled such that the mask spanned the lower edge of the chin, the bridge of the nose, and the edges of the cheeks.

As depicted in Figure 1B, each face stimulus was preceded by a sentence. In the Context condition, the sentence described a face expression within a congruent happy or sad emotional event happening to the protagonists (e.g., “Her pet cat was *found/lost* yesterday afternoon”). Male and female happy and sad variations of 12 emotional sentence themes from McCrackin and Itier (2021) were presented,^5^ as they have repeatedly been demonstrated to elicit the expected emotional responses (McCrackin and Itier, 2021; McCrackin et al., 2022b). In the No Context condition, the sentence “Get ready for the next person to appear” was displayed. This sentence had the same number of syllables as the emotional sentences and similarly referenced the protagonist but did not provide any emotional information.

### Design and procedure

The study was a repeated measures design with three factors---Context (2; Context; No Context), Emotion (2; Happy, Sad), and Mask (2; Mask, No Mask). Context manipulated whether an emotional sentence (Context) or neutral sentence (No Context) preceded the presentation of a protagonist’s emotional face. This variable was blocked, such that half of the testing blocks (i.e., 4) provided emotional context (Context blocks) and half did not (No Context blocks), with the block order and trials within the blocks randomized. The factor of Emotion manipulated whether the face depicted a Happy or Sad facial expression. Facial expression and emotional context were congruent during the Context condition such that happy context sentences were always paired with happy expressions and sad context sentences were paired with sad expressions.^6^ The Mask factor manipulated whether the face wore a face mask (Mask) or not (No Mask).

Manipulating these three factors yielded 8 experimental conditions. Each condition was sampled 24 times for a total of 192 trials divided across 8 testing blocks. The same face identities were presented in the Context and No Context blocks so that the impact of emotional context could be examined without changing any other variables (i.e., participants saw the same face image once with context and once without context). All conditions were equiprobable and presented using a pseudorandom sequence.

Figure 1B illustrates a typical trial. Participants were first shown either an emotional context sentence (Context condition) or a neutral sentence (No Context condition) for 4,000 ms. A 200 ms fixation cross preceded a presentation of the Happy or Sad emotional face either wearing a mask (Mask) or not wearing a mask (No Mask) for 2,000 ms. After the image presentation, participants were asked to use a 9-point Likert scale to rate the protagonists’ *(i)* emotional intensity ranging from 0/very un-intense to 9/very intense, and *(ii)* emotional valence from 0/very negative to 9/very positive on separate screens and were given unlimited time to make each response. These two rating scales were designed to probe the affective dimensions of valence (pleasure) and arousal (intensity) theorized by the Circumplex model of emotion (Russell, 1980) to represent dissociated components of the emotional experience.

## Results

Mean ratings of the protagonists’ emotional intensity and valence were calculated for each participant. Then, two separate repeated measures ANOVAs were run on each dependant variable (i.e., intensity and valence) with Context (Context, No Context), Emotion (Happy, Sad), and Mask (Mask, No mask) included as factors. Follow-up two-tailed paired-test tests were performed where required, with Bonferroni correction applied to the nominal α = 0.05 level.

To remind, we hypothesized that if context contributes significantly to emotional understanding, availability of a congruent emotional context should provide mitigating effect under conditions in which visual facial cues are unavailable due to facial obstruction by mask.

### Intensity

**FIGURE 2:**
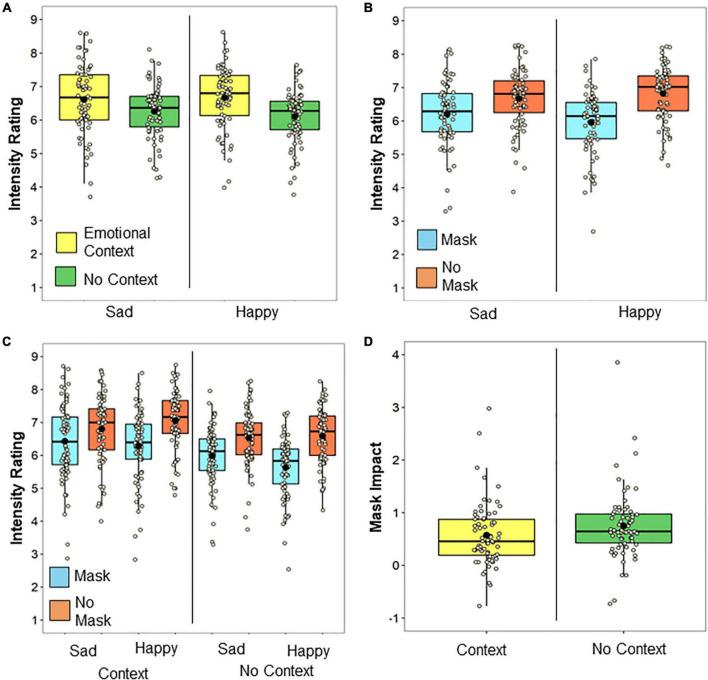
Intensity ratings. The mean and median for each condition are indicated with a black dot and solid line, respectively, and each participants’ data point is plotted. Impact of **(A)** emotional context and **(B)** mask on intensity ratings for each emotion. **(C)** The impact of masks on intensity ratings as a function of Emotion and Context. **(D)** The overall mask impact (unmasked intensity—masked intensity ratings) with and without context. Note that the rating of 1 represents the lowest emotional intensity.

Confirming the efficacy of our context manipulation, a main effect of Context indicated that intensity ratings were overall higher when a protagonist was presented within an emotional context relative to no context [*F*(1, 69) = 48.92, *MSE* = 0.60, *p* = 1.36 × 10^–9^, η*_*p*_^2^* = 0.42]. As depicted in Figure 2A, a Context × Emotion interaction indicated that availability of an emotional context increased intensity ratings for happy emotions more than for sad ones [*F*(1, 69) = 7.87, *MSE* = 0.18, *p* < 0.007, η*_*p*_*^2^ = 0.10], although the effect was significant for both happy [*t*(69) = 8.13, *p* = 1.14 × 10^–11^, *SE* = 0.080 *d* = 0.97] and sad trials [*t*(69) = 4.46, *p* = 3.1 × 10^–5^, *SE* = *0.069, d* = 0.53].

Replicating previous work indicating that facial obstruction by masks alters emotional inferences (McCrackin et al., 2022b), a main effect of Mask indicated that faces wearing masks were judged to feel less intense emotion than those not wearing masks [*F*(1, 69) = 84.87, *MSE* = 0.73, *p* = 1.23 × 10^–13^, η*_*p*_*^2^ = 0.55]. As shown in Figure 2B, a Mask x Emotion interaction indicated that this reduction in perceived emotional intensity for faces wearing masks was also larger for happy than for sad emotions [*F*(1, 69) = 38.30, *MSE* = 0.15, *p* = 3.80 × 10^–8^, η*_*p*_*^2^ = 0.36], although wearing masks reduced emotional intensity ratings for both happy [*t*(69) = −10.35, *p* = 1.09 × 10^–15^, *SE* = 0.083, *d* = −1.24] and sad emotions [*t*(69) = −6.18, *p* = 3.92 × 10^–8^, *SE* = 0.074, *d* = −0.74].

Finally, we predicted that adding emotional context may be able to reduce some of the negative impact of facial occlusion by masks on emotional inferences. Indeed, and as depicted in Figure 2C, there was a significant Context × Mask interaction [*F*(1, 69) = 16.39, *MSE* = 0.065, *p* = 1.33 × 10^–4^, η*_*p*_*^2^ = 0.19]. While there was an effect of mask for both Context [*t*(69) = −7.93, *p* = 2.68 × 10^–11^, *SE* = 0.072, *d* = −0.95] and No Context conditions [*t*(69) = 9.67, *p* = 1.81 × 10^–14^, *SE* = 0.072, *d* = −1.16], the effect of visual occlusion by Masks (computed as Intensity rating without masks—Intensity rating with masks) was reduced by approximately 23% when Context was provided [*t*(69) = −4.05, *p* = 1.33 × 10^–4^, *SE* = 0.043, *d* = −0.48], as depicted in Figure 2D. The three-way interaction between Context, Emotion, and Mask was not significant [*F*(1, 69) = 0.005, MSE = 0.08, *p* = 0.94, η*_*p*_^2^* < 0.001].

### Valence

Similarly to intensity, and confirming the efficacy of the emotional manipulation, a main effect of Emotion valence indicated that protagonists were judged to feel more positive in the happy emotional condition compared to the sad emotional condition [*F*(1, 69) = 1537.32, *MSE* = 1.97, *p* = 6.75 × 10^–49^, η*_*p*_*^2^ = 0.96]. A main effect of Mask reflected that protagonists were also judged to feel more positive when their face was visually unobstructed as opposed to when they were wearing masks [*F*(1, 69) = 33.29, *MSE* = 0.11, *p* = 2.06 × 10^–7^, η*_*p*_*^2^ = 0.33], although this was qualified by a Mask by Emotion interaction discussed below.

**FIGURE 3:**
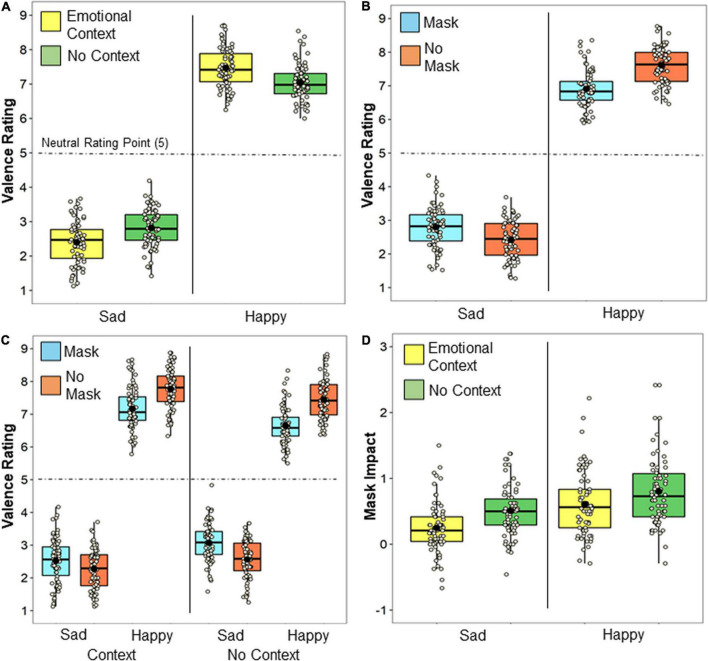
Valence ratings. The mean and median for each condition are indicated with a black dot and solid line, respectively, and each participants’ data point is plotted. Impact of **(A)** emotional context and **(B)** mask on valence ratings for each emotion. **(C)** The impact of masks on valence ratings as a function of emotion and context. **(D)** The overall impact of masks (unmasked valence—masked valence ratings for happy emotion, and masked valence—unmasked valence for sad emotion) for positive happy and sad negative emotions as a function of context. The neutral valence point (rating 5) is indicated by the dotted line.

As shown in Figure 3A, significant Context x Emotion interaction indicated that availability of emotional context led to heightened assumptions of the individual feeling the inferred emotion [*F*(1, 69) = 94.41, *MSE* = 0.26, *p* = 1.51 × 10^–14^, η*_*p*_*^2^ = 0.58]. That is, availability of an emotional context increased valence ratings for happy trials (*p* < *0.001*) and decreased valence ratings for sad trials (*p* < *0.001*), with the magnitude of this effect no different between the happy and sad emotion conditions (*p* = 0.86).

Further, as depicted in Figure 3B, a Mask x Emotion interaction [*F*(1, 69) = 41.06, *MSE* = 0.22, *p* = 2.50 × 10^–21^, η*_*p*_*^2^ = 0.73] indicated that individuals wearing masks were rated as feeling more neutral emotions than those not wearing masks, replicating our previous work (McCrackin et al., 2022b). For happy trials, protagonists wearing masks were rated with lower valence ratings [*t*(69) = −12.77, *p* = 7.58 × 10^–20^, *SE* = 0.055, *d* = −0.1.53], while for sad trials, they were rated with higher valence ratings [*t*(69) = 9.18, *p* = 1.43 × 10^–13^, *SE* = 0.042, *d* = 1.10]. The impact of masks on valence ratings was larger for happy trials than for sad trials [*t*(69) = 5.77, *p* = 2.06 × 10^–7^, *SE* = 0.057, *d* = 0.69].

Finally, as illustrated in Figure 3C, there was also a three-way interaction between Context, Emotion, and Mask [*F*(1, 69) = 48.74, *MSE* = 0.04, *p* = 1.44 × 10^–9^, η*_*p*_*^2^ = 0.41]. As shown, this interaction was driven by the availability of emotional context reducing the impact of masks on valence ratings during both sad [*t*(69) = −9.26, *p* = 1.27 × 10^–13^, *SE* = 0.045, *d* = −1.11] and happy [*t*(69) = 8.76, *p* = 8.30 × 10^–13^, *SE* = 0.048, *d* = 1.05] emotions, with a similar magnitude [*t*(69) = 0.17, *p* = 0.86, *SE* = 0.036, *d* = 0.02]. Proportionately, availability of context reduced the negative effect of facial occlusion on valence ratings, plotted in Figure 3D, by 51% for sad emotions and by 24% for happy emotions.

To summarize, providing emotional context was overall associated with emotional judgments of protagonists feeling more intense emotions and stronger emotional valence. When protagonists wore face masks they were judged as feeling less intense and weaker emotional valence, particularly for happy emotions. The availability of emotional context significantly reduced the negative impact of face masks on both ratings of intensity and valence, with the magnitude of this reduction ranging from 23% up to 51%.

## Discussion

Lower face occlusion with face masks has been shown to impair our ability to recognize facial emotional expressions (Carbon, 2020; Grundmann et al., 2021; Carbon and Serrano, 2021; Williams et al., 2021; Grenville and Dwyer, 2022; Kim et al., 2022; McCrackin et al., 2022a), prompting concerns about the effectiveness of social interactions in masked situations (Mheidly et al., 2020; Molnar-Szakacs et al., 2021). However, during real life social interactions, emotional expressions are typically experienced within a broader emotional context that might compensate for the lack of lower face cues (Wieser and Brosch, 2012). Thus, it is important to consider how emotional context may affect emotional processing under conditions when visual emotional information from faces may not be available.

To investigate this question, we presented participants with images of emotional faces who either wore masks or had their faces visually unobstructed. Critically, the protagonists were presented within either an emotional or neutral context. Participants were asked to rate the emotional state of each protagonist. The data indicated overall reduced emotional processing from faces wearing masks. Availability of emotional context reduced, but did not fully reverse, this negative impact of facial occlusion. Next, we discuss two points relating to these data.

First, replicating and extending existing reports (Carbon, 2020 for a review) we found an impact of face occlusion by masks on emotional inferences, both when emotional context was available and when it was not available. When protagonists wore masks they were judged as feeling more neutral and less intense emotion. This finding dovetails with recent work from both emotion recognition (Carbon, 2020; Grundmann et al., 2021; Carbon and Serrano, 2021; Williams et al., 2021; Grenville and Dwyer, 2022; Kim et al., 2022; McCrackin et al., 2022a) and emotional valence and intensity paradigms (McCrackin et al., 2022b) to suggest that face occlusion by masks significantly impacts not only basic emotion recognition but also judgments of emotional states that integrate both emotional expressions and contextual information.

Of note here is our finding that face covering by masks seemed to impact happy emotional inferences more than sad ones. We recently found a similar asymmetry for understanding happy emotions to be more impacted than understanding sad emotions when we asked participants to both infer and share emotions with protagonists wearing face masks (McCrackin et al., 2022b). One explanation for this finding is that face masks impact the perception of happy expressions more than they impair the perception of sad ones. Consequently, this basic emotion perception impairment may exert a larger downstream effect on happy emotional inferences. While some recent studies have reported greater impact in recognizing sad relative to happy expressions from masked faces (Carbon, 2020; Williams et al., 2021; Kim et al., 2022; McCrackin et al., 2022a), it is important to highlight that these studies have mainly examined overall emotion recognition accuracy (i.e., percent correct identifications) and happy facial expressions are well known to be the easiest emotion to recognize (i.e., the so-called happy superiority effect in emotion recognition; e.g., Neath, 2012; for a review; Svard et al., 2012; Švegar et al., 2013). It is possible that happy expressions are still easily recognized from faces wearing masks due to ceiling effects, but the perceived intensity and valence of those happy expressions remain more strongly impacted than perceived intensity and valence of sad expressions. In line with this point, our data also suggest that perception of happy facial expressions was impacted more by lower face occlusion than the perception of sad facial expressions. This is likely because the diagnostic smile is fully covered by the face mask, while the eyes remain unobstructed as a clear diagnostic feature for sadness (e.g., Ekman, 1999; Smith et al., 2005; Blais et al., 2012; Kret and de Gelder, 2012; Wegrzyn et al., 2017). Future studies are needed to further understand the links between different facets of emotional inferences under conditions in which facial cues may not be readily available.

Second, we also found that availability of an emotional context reduced the negative impact of facial occlusion by masks on ratings of both happy and sad emotional states. That is, masked individuals received closer ratings to the unmasked individuals when their images were paired with a congruent emotional context as opposed to when their images were paired with a neutral context. Thus, while contextual information was not necessary for understanding the general emotional state, it modulated the extent to which the inferred emotional state was impacted by visual occlusion of face parts. The reduction of the mask impact ranged from 23% for intensity ratings, and 51% for sad valence ratings, to 24% for happy valence ratings. As such, this suggests that providing contextual statements during social interactions while protagonists wear masks may provide a relatively simple way in which the impact of facial occlusion by masks can be reduced.

There are a few points that warrant further investigation. First, our study used static images with photoshopped masks. Since dynamicity typically facilitates emotion recognition (e.g., Weyers et al., 2006; Enticott et al., 2014; but see Gold et al., 2013), dynamic emotional stimuli may facilitate or change emotion recognition while protagonists wear masks. It is also possible that actors wearing masks may change how they emote with their upper face features, as suggested by Okazaki et al. (2021). Future work is needed to understand the commonalities and differences in emotional communication in static and dynamic experimental conditions. Second, here we focused on understanding how facial occlusion and emotional context impacted understanding of happy and sad emotional expressions, but previous work has shown that face masks impair the perception of all six basic emotions (e.g., McCrackin et al., 2022a). An interesting next step would be to investigate whether availability of a congruent context can also reduce the impact of masks on recognition of other basic emotions as well. It is possible that understanding emotions with diagnostic upper face features (e.g., fear) may not be as impacted by contextual manipulations as understanding emotions with diagnostic lower face features (e.g., disgust) when lower visual features are occluded by masks. Opposite results may be expected for eye coverings. Third, we focused on the use of face masks as visual occluders, but there is evidence to suggest that the type of face occlusion may matter (Wang et al., 2015). For example, Fischer et al. (2012) reported that covering the lower face with a niqab led to a bias to perceive emotions as more negative, while Kret and Fischer (2018) reported key differences in how emotions were recognized when lower faces were covered by a western winter scarf relative to a niqab. Face masks themselves may now have implied positive or negative responses depending on the participant and their experiences, so future studies can examine the role of the type of face covering in social attribution effects. Finally, individual participant factors may have also played a role in our results. For example, our sample was mostly western, female skewed, and educated young adults. Individual factors such as gender (e.g., Hoffmann et al., 2010; Abbruzzese et al., 2019; Gamsakhurdashvili et al., 2021), age (Abbruzzese et al., 2019; Carbon, 2020), and cultural experience (Elfenbein et al., 2002) have been shown to play a role in emotion recognition. For example, women appear to be better at detecting subtle facial emotions (Hoffmann et al., 2010), and thus our participants may have better emotions recognition overall (from masked and unmasked faces). An important next step would be to examine if these results generalize to a more diverse sample.

In summary, wearing face masks lowers our ability to infer emotional states in others, with inferences about happy emotional states affected more than inferences about sad emotional states. This negative impact of visual occlusion by face masks can be reduced by incorporating verbal statements which provide congruent emotional context.

## Data availability statement

The datasets presented in this study can be found in online repositories. The names of the repository/repositories and accession number(s) can be found below: https://osf.io/9bmr3/?view_only=e0871f7add364e378eccd9920f60d98b.

## Ethics statement

The studies involving human participants were reviewed and approved by the McGill University REB. The patients/participants provided their written informed consent to participate in this study. Written informed consent was obtained from the individual(s) for the publication of any identifiable images or data included in this article.

## Author contributions

SM and JR were involved in the early conceptualization and experimental design. SM oversaw the study programming and carried out data processing and analysis with advice from JR. SM wrote the initial manuscript draft, which was later revised and approved by both authors.

## Footnotes

1
https://osf.io/hm59u

2 Data quality was very high. No participants met our pre-registered exclusions criteria of having more than 20% data loss due to anticipations (responses faster than 500 ms) or not providing responses. Data from 71 participants was originally collected, but one participant was removed from analysis because there was a record of them completing the study multiple times. In the final sample, trial completion was high, with the average number of valence and arousal responses completed being 191.79/192 (*SD* = 0.70) and 192.93/192 (*SD* = 0.31), respectively. All participants had normal or corrected-to-normal vision, English fluency, no diagnosis of mental illness, and no previous head trauma.

3
https://www.testable.org/

4 Stimuli Identities: Males- KM08, KM11, KM31, M13, M25, M16, M37, M49, M57, M62, M66, M72, M81, M89, M105, M99, M109, M114, M119, M123. Females—F48, F54, F63, F69, F71, F85, F90, F98, F101, F106, F115, F125, F132, F134, F140, F162, F171, F163, F177, F182.

5 The following sentence themes were selected from the list of 25 starred themes in McCrackin and Itier (2021, Table 1), with Theme 1 referencing the first starred sentence: Theme 13, Theme 14, Theme 15, Theme 16, Theme 17, Theme 18, Theme 20, Theme 21, Theme 22, Theme 23, Theme 24, Theme 25.

6 We had examined how a neutral emotion condition (using both neutral sentences and facial expression) influences affective judgments in our recent work assessing how face covering with opaque vs. transparent face masks impacted affective theory of mind and empathy ratings (McCrackin et al., 2022b). In this study, we observed that the neutral emotion condition generated data which fell in between happy and sad conditions (as it would be expected), with ratings close a perfect neutral score of 5 on the Likert scale when faces were covered by a transparent mask. Given this result, and to maximize understanding the differences in emotional processing for positively and negatively valanced emotions, in the present study we examined only the positive and negative emotional conditions.

## References

[B1] Abbruzzese L. Magnani N. Robertson I. H. Mancuso M. (2019). Age and gender differences in emotion recognition. *Front. Psychol.* 10:2371. 10.3389/fpsyg.2019.02371 31708832 PMC6819430

[B2] Addington J. Saeedi H. Addington D. (2006). Facial affect recognition: A mediator between cognitive and social functioning in psychosis?. *Schizoph. Res.* 85 142–150. 10.1016/j.schres.2006.03.028 16678388

[B3] Anderson A. K. Sobel N. (2003). Dissociating intensity from valence as sensory inputs to emotion. *Neuron* 39, 581–583. 12925272 10.1016/s0896-6273(03)00504-x

[B4] Baron-Cohen S. Cross P. (1992). Reading the eyes: Evidence for the role of perception in the development of a theory of mind. *Mind Lang.* 7 172–186. 10.1111/j.1468-0017.1992.tb00203.x

[B5] Besel L. D. Yuille J. C. (2010). Individual differences in empathy: The role of facial expression recognition. *Pers. Individ. Differ.* 49 107–112. 10.1016/j.paid.2010.03.013

[B6] Biermann M. Schulze A. Unterseher F. Atanasova K. Watermann P. Krause-Utz A. (2021). Trustworthiness appraisals of faces wearing a surgical mask during the Covid-19 pandemic in Germany: An experimental study. *PLoS One* 16:e0251393. 10.1371/journal.pone.0251393 34003836 PMC8130962

[B7] Blais C. Roy C. Fiset D. Arguin M. Gosselin F. (2012). The eyes are not the window to basic emotions. *Neuropsychologia* 50 2830–2838. 10.1016/j.neuropsychologia.2012.08.010 22974675

[B8] Carbon C. C. (2020). Wearing face masks strongly confuses counterparts in reading emotions. *Front. Psychol.* 11:566886. 10.3389/fpsyg.2020.566886 33101135 PMC7545827

[B9] Carbon C. C. Serrano M. (2021). The Impact of Face Masks on the Emotional Reading Abilities of Children—A Lesson From a Joint School–University Project. *IPerception* 12:20416695211038265. 10.1177/20416695211038265 34447567 PMC8383324

[B10] Cartaud A. Quesque F. Coello Y. (2020). Wearing a face mask against Covid-19 results in a reduction of social distancing. *PLoS One* 15:e0243023. 10.1371/journal.pone.0243023 33284812 PMC7721169

[B11] Clark T. F. Winkielman P. McIntosh D. N. (2008). Autism and the extraction of emotion from briefly presented facial expressions: Stumbling at the first step of empathy. *Emotion* 8:803. 10.1037/a0014124 19102591

[B12] Colibazzi T. Posner J. Wang Z. Gorman D. Gerber A. Yu S. (2010). Neural systems subserving valence and arousal during the experience of induced emotions. *Emotion* 10: 377. 10.1037/a0018484 20515226

[B13] Decety J. Lewis K. L. Cowell J. M. (2015). Specific electrophysiological components disentangle affective sharing and empathic concern in psychopathy. *J. Neurophysiol.* 114 493–504. 10.1152/jn.00253.2015 25948868 PMC4509400

[B14] Ebner N. C. Riediger M. Lindenberger U. (2010). FACES—A database of facial expressions in young, middle-aged, and older women and men: Development and validation. *Behav. Res. Methods* 42 351–362. 10.3758/BRM.42.1.351 20160315

[B15] Eikenberry S. E. Mancuso M. Iboi E. Phan T. Eikenberry K. Kuang Y. (2020). To mask or not to mask: Modeling the potential for face mask use by the general public to curtail the COVID-19 pandemic. *Infect. Dis. Model.* 5 293–308. 10.1016/j.idm.2020.04.001 32355904 PMC7186508

[B16] Ekman P. (1999). Basic emotions. *Handb. Cogn. Emot.* 98:16.

[B17] Elfenbein H. A. Mandal M. K. Ambady N. Harizuka S. Kumar S. (2002). Cross-cultural patterns in emotion recognition: Highlighting design and analytical techniques. *Emotion* 2:75. 10.1037/1528-3542.2.1.75 12899367

[B18] Enticott P. G. Kennedy H. A. Johnston P. J. Rinehart N. J. Tonge B. J. Taffe J. R. (2014). Emotion recognition of static and dynamic faces in autism spectrum disorder. *Cogn. Emot.* 28 1110–1118. 10.1080/02699931.2013.867832 24341852

[B19] Fischer A. H. Gillebaart M. Rotteveel M. Becker D. Vliek M. (2012). Veiled emotions: The effect of covered faces on emotion perception and attitudes. *Soc. Psychol. Pers. Sci.* 3 266–273. 10.1177/1948550611418534

[B20] Gabrieli G. Esposito G. (2021). Reduced Perceived Trustworthiness during Face Mask Wearing. *Eur. J. Investig. Health Psychol. Educ.* 11 1474–1484. 10.3390/ejihpe11040105 34842683 PMC8628681

[B21] Gamsakhurdashvili D. Antov M. I. Stockhorst U. (2021). Facial Emotion Recognition and Emotional Memory From the Ovarian-Hormone Perspective: A Systematic Review. *Front. Psychol.* 12:641250. 10.3389/fpsyg.2021.641250 34093322 PMC8174660

[B22] Goeleven E. De Raedt R. Leyman L. Verschuere B. (2008). The Karolinska directed emotional faces: A validation study. *Cogn. Emot.* 22 1094–1118. 10.1080/02699930701626582 29312053

[B23] Gold J. M. Barker J. D. Barr S. Bittner J. L. Bromfield W. D. Chu N. (2013). The efficiency of dynamic and static facial expression recognition. *J. Vis.* 13 23–23. 10.1167/13.5.23 PMC3666543 23620533

[B24] Grenville E. Dwyer D. M. (2022). Face masks have emotion-dependent dissociable effects on accuracy and confidence in identifying facial expressions of emotion. *Cogn. Res.* 7:15. 10.1186/s41235-022-00366-w 35157157 PMC8844328

[B25] Grundmann F. Epstude K. Scheibe S. (2021). Face masks reduce emotion-recognition accuracy and perceived closeness. *PLoS One* 16:e0249792. 10.1371/journal.pone.0249792 33891614 PMC8064590

[B26] Hies O. Lewis M. B. (2022). Beyond the beauty of occlusion: Medical masks increase facial attractiveness more than other face coverings. *Cogn. Res.* 7:1. 10.1186/s41235-021-00351-9 35006366 PMC8743690

[B27] Hoffmann H. Kessler H. Eppel T. Rukavina S. Traue H. C. (2010). Expression intensity, gender and facial emotion recognition: Women recognize only subtle facial emotions better than men. *Acta Psychol.* 135 278–283. 10.1016/j.actpsy.2010.07.012 20728864

[B28] Hugenberg K. Wilson J. P. (2013). “Faces are central to social cognition,” in *The Oxford handbook of social cognition*, ed. Carlston D. E. (Oxford: Oxford University Press), 167–193. 10.1093/oxfordhb/9780199730018.013.0009

[B29] Kessinger E. A. Corkin S. (2004). Two routes to emotional memory: Distinct neural processes for valence and arousal. *Proc. Natl. Acad. Sci. U.S.A*. 101, 3310–3315. 14981255 10.1073/pnas.0306408101 PMC365786

[B30] Kim G. Seong S. H. Hong S. S. Choi E. (2022). Impact of face masks and sunglasses on emotion recognition in South Koreans. *PLoS One* 17:e0263466. 10.1371/journal.pone.0263466 35113970 PMC8812856

[B31] Klapper A. Dotsch R. van Rooij I. Wigboldus D. H. (2016). Do we spontaneously form stable trustworthiness impressions from facial appearance? *J. Pers. Soc. Psychol.* 111:655. 10.1037/pspa0000062 27762574

[B32] Kret M. de Gelder B. (2012). Islamic headdress influences how emotion is recognized from the eyes. *Front. Psychol.* 3:110. 10.3389/fpsyg.2012.00110 22557983 PMC3322610

[B33] Kret M. E. Fischer A. H. (2018). Recognition of facial expressions is moderated by Islamic cues. *Cogn. Emot.* 32 623–631. 10.1080/02699931.2017.1330253 28566058

[B34] Leppänen J. M. Hietanen J. K. (2001). Emotion recognition and social adjustment in school–aged girls and boys. *Scand. J. Psychol.* 42 429–435. 10.1111/1467-9450.00255 11771812

[B35] Leung N. H. Chu D. K. Shiu E. Y. Chan K. H. McDevitt J. J. Hau B. J. (2020). Respiratory virus shedding in exhaled breath and efficacy of face masks. *Nat. Med.* 26 676–680. 10.1038/s41591-020-0843-2 32371934 PMC8238571

[B36] Lundqvist D. Flykt A. Öhman A. (1998). Karolinska directed emotional faces. *Cogn. Emot.* 22 1094–1118. 10.1037/t27732-000

[B37] Marsh A. A. Kozak M. N. Ambady N. (2007). Accurate identification of fear facial expressions predicts prosocial behavior. *Emotion* 7:239. 10.1037/1528-3542.7.2.239 17516803 PMC2743452

[B38] McCrackin S. D. Capozzi F. Mayrand F. Ristic J. (2022a). Face masks impair basic emotion recognition: Group effects and individual variability. *Soc. Psychol.* [Epub ahead of print]. 10.1027/1864-9335/a000470

[B39] McCrackin S. D. Provencher S. Mendell E. Ristic J. (2022b). Transparent masks reduce the negative impact of opaque masks on understanding emotional states but not on sharing them. *Cogn. Res.* 7:59. 10.1186/s41235-022-00411-8 35796906 PMC9261140

[B40] McCrackin S. D. Itier R. J. (2021). Feeling through another’s eyes: Perceived gaze direction impacts ERP and behavioural measures of positive and negative affective empathy. *NeuroImage* 226:117605. 10.1016/j.neuroimage.2020.117605 33271267

[B41] Mheidly N. Fares M. Y. Zalzale H. Fares J. (2020). Effect of face masks on interpersonal communication during the COVID-19 pandemic. *Front. Public Health* 8:582191. 10.3389/fpubh.2020.582191 33363081 PMC7755855

[B42] Mier D. Lis S. Neuthe K. Sauer C. Esslinger C. Gallhofer B. (2010). The involvement of emotion recognition in affective theory of mind. *Psychophysiology* 47 1028–1039. 10.1111/j.1469-8986.2010.01031.x 20456660

[B43] Molnar-Szakacs I. Uddin L. Q. Heffernan M. B. (2021). The face behind the mask: The future of interpersonal interaction. *Neuron* 109 1918–1920. 10.1016/j.neuron.2021.05.030 34139182 PMC8730492

[B44] Neath K. (2012). *The use of facial features in facial expression discrimination.* Ph.D thesis, Waterloo: University of Waterloo.

[B45] Okazaki S. Yamanami H. Nakagawa F. Takuwa N. Kawabata Duncan K. J. (2021). Mask wearing increases eye involvement during smiling: A facial EMG study. *Sci. Rep.* 11:20370. 10.1038/s41598-021-99872-y 34645906 PMC8514576

[B46] Parada-Fernández P. Herrero-Fernández D. Jorge R. Comesaña P. (2022). Wearing mask hinders emotion recognition, but enhances perception of attractiveness. *Pers. Individ. Differ.* 184:111195. 10.1016/j.paid.2021.111195 PMC9755824 36540665

[B47] Prather K. A. Wang C. C. Schooley R. T. (2020). Reducing transmission of SARS-CoV-2. *Science* 368 1422–1424. 10.1126/science.abc6197 32461212

[B48] Russell J. A. (1980). A circumplex model of affect. *J. Pers. Soc. Psychol*. 39:1161.

[B49] Smith M. L. Cottrell G. W. Gosselin F. Schyns P. G. (2005). Transmitting and decoding facial expressions. *Psychol. Sci.* 16 184–189. 10.1111/j.0956-7976.2005.00801.x 15733197

[B50] Stewart S. L. Schepman A. Haigh M. McHugh R. Stewart A. J. (2019). Affective theory of mind inferences contextually influence the recognition of emotional facial expressions. *Cogn. Emot.* 33 272–287. 10.1080/02699931.2018.1450224 29540095

[B51] Svard J. Wiens S. Fischer H. (2012). Superior recognition performance for happy masked and unmasked faces in both younger and older adults. *Front. Psychol.* 3:520. 10.3389/fpsyg.2012.00520 23226135 PMC3510469

[B52] Švegar D. Kardum I. Poliè M. (2013). Happy face superiority effect in change detection paradigm. *Psihol. Teme* 22 249–269.

[B53] Wang Y. Thomas J. Weissgerber S. C. Kazemini S. Ul-Haq I. Quadflieg S. (2015). The headscarf effect revisited: Further evidence for a culture-based internal face processing advantage. *Perception* 44 328–336. 10.1068/p7940 26562256

[B54] Wegrzyn M. Vogt M. Kireclioglu B. Schneider J. Kissler J. (2017). Mapping the emotional face. How individual face parts contribute to successful emotion recognition. *PLoS One* 12:e0177239. 10.1371/journal.pone.0177239 28493921 PMC5426715

[B55] Weyers P. Mühlberger A. Hefele C. Pauli P. (2006). Electromyographic responses to static and dynamic avatar emotional facial expressions. *Psychophysiology* 43 450–453. 10.1111/j.1469-8986.2006.00451.x 16965606

[B56] Wieser M. J. Brosch T. (2012). Faces in context: A review and systematization of contextual influences on affective face processing. *Front. Psychol.* 3:471. 10.3389/fpsyg.2012.00471 23130011 PMC3487423

[B57] Williams T. A. Porter M. A. Langdon R. (2014). Social approach and emotion recognition in fragile X syndrome. *Am. J. Intellect. Dev. Disabil.* 119 133–150. 10.1352/1944-7558-119.2.133 24679350

[B58] Williams W. C. Haque E. Mai B. Venkatraman V. (2021). Face masks influence how facial expressions are perceived: A drift-diffusion model of emotion judgments. *PsyArXiv* [Preprint]. 10.31234/osf.io/a8yxf PMC10231301 37258558

